# Deep learning model to identify and validate hypotension endotypes in surgical and critically ill patients

**DOI:** 10.1016/j.bja.2024.10.048

**Published:** 2025-01-08

**Authors:** Zhongping Jian, Xianfu Liu, Karim Kouz, Jos J. Settels, Simon Davies, Thomas W.L. Scheeren, Neal W. Fleming, Denise P. Veelo, Alexander P.J. Vlaar, Michael Sander, Maxime Cannesson, David Berger, Michael R. Pinsky, Daniel I. Sessler, Feras Hatib, Bernd Saugel

**Affiliations:** 1 Edwards Lifesciences, Irvine, CA, USA; 2 Department of Anesthesiology, Center of Anesthesiology and Intensive Care Medicine, University Medical Center Hamburg-Eppendorf, Hamburg, Germany; 3 Outcomes Research Consortium, Cleveland, OH, USA; 4 Centre for Health and Population Sciences, Hull York Medical School, University of York, York, UK; 5 Department of Anesthesiology & Pain Medicine, UC Davis School of Medicine, Sacramento, CA, USA; 6 Amsterdam UMC, University of Amsterdam, Department of Anesthesiology, Amsterdam, The Netherlands; 7 Amsterdam UMC, University of Amsterdam, Department of Intensive Care Medicine, Amsterdam, The Netherlands; 8 Department of Anaesthesiology, Intensive Care Medicine and Pain Medicine, University Hospital Giessen, Justus-Liebig University Giessen, Giessen, Germany; 9 Department of Anesthesiology and Perioperative Medicine, David Geffen School of Medicine at UCLA, Los Angeles, CA, USA; 10 Department of Intensive Care Medicine, Inselspital, Bern University Hospital, Bern University, Bern, Switzerland; 11 Department of Critical Care Medicine, University of Pittsburgh, Pittsburgh, PA, USA; 12 Department of Anesthesiology and Center for Outcomes Research, University of Texas Health Sciences Center, Houston, TX, USA

**Keywords:** anaesthesia, blood pressure, cardiac output, haemodynamic monitoring, hypotension, machine learning

## Abstract

**Background:**

Hypotension is associated with organ injury and death in surgical and critically ill patients. In clinical practice, treating hypotension remains challenging because it can be caused by various underlying haemodynamic alterations. We aimed to identify and independently validate endotypes of hypotension in big datasets of surgical and critically ill patients using unsupervised deep learning.

**Methods:**

We developed an unsupervised deep learning algorithm, specifically a deep learning autoencoder model combined with a Gaussian mixture model, to identify endotypes of hypotension based on stroke volume index, heart rate, systemic vascular resistance index, and stroke volume variation observed during episodes of hypotension. The algorithm was developed with data from 871 surgical patients who had 6962 hypotensive events and validated in two independent datasets, one including 1000 surgical patients who had 7904 hypotensive events and another including 1000 critically ill patients who had 53 821 hypotensive events. We defined hypotension as a mean arterial pressure <65 mm Hg for at least 1 min.

**Results:**

In the development dataset, we identified four hypotension endotypes. Based on their physiological and clinical characteristics, we labelled them as: vasodilation, hypovolaemia, myocardial depression, and bradycardia. The same four hypotension endotypes were identified in the two independent validation datasets of surgical and critically ill patients.

**Conclusions:**

Unsupervised deep learning identified four endotypes of hypotension in surgical and critically ill patients: vasodilation, hypovolaemia, myocardial depression, and bradycardia. The algorithm provides the probability of each endotype for each hypotensive data point. Identifying hypotensive endotypes could guide clinicians to causal treatments for hypotension.

**Editor's key points**

• Hypotension in surgical and critically ill patients occurs because of various underlying haemodynamic changes that respond to distinct treatments based on their causal mechanisms.
• The authors used an unsupervised deep learning algorithm to identify endotypes of hypotension based on stroke volume index, heart rate, systemic vascular resistance index, and stroke volume variation observed during episodes of hypotension in 871 surgical patients.
• The algorithm identified four hypotension endotypes. Based on their physiological and clinical characteristics, these were labelled as: vasodilation, hypovolaemia, myocardial depression, and bradycardia. The endotypes were validated in two independent datasets of 1000 surgical patients and 1000 critically ill patients.
• The algorithm provides the probability of each endotype for each hypotensive event, which might guide clinicians to mechanism-based treatments for hypotension.

Hypotension is associated with organ injury and death in patients having surgery^1^, ^2^, ^3^ and in critically ill patients.^4^, ^5^, ^6^ Clinicians thus try to avoid hypotension. However, avoiding and treating hypotension can be challenging because the underlying haemodynamic alterations are often unclear. A better understanding of the root causes of hypotension could allow hypotension to be prevented or treated with specific mechanism-based targeted interventions.

However, understanding the causes of hypotension at the bedside in real time is challenging, as haemodynamic variables closely interact, rapidly change, and are influenced by therapeutic interventions. Machine learning can process large amounts of data in a short time and might thereby be used to help clinicians identify causes of hypotension. In a single-centre pilot study, traditional unsupervised machine learning (hierarchical clustering) identified six endotypes of hypotension in 100 patients having major abdominal surgery.^7^ It was concluded that these endotypes of hypotension must be confirmed in larger and more heterogenous patient populations before endotypes can be recommended to causally treat hypotension.^7^ We therefore aimed to identify and independently validate endotypes of hypotension in big datasets of surgical and critically ill patients using unsupervised deep learning.

## Methods

We conducted a retrospective analysis of three aggregated datasets including prospectively collected anonymised data of a total of 2871 surgical or critically ill patients. The development dataset included 871 surgical patients from six previously reported studies.^8^, ^9^, ^10^, ^11^, ^12^, ^13^ The first independent validation dataset included 1000 surgical patients, and the second independent validation dataset included 1000 critically ill patients. The datasets are described in the Supplementary material and patient characteristics are shown in Supplementary Table S1.

### Haemodynamic monitoring data used for endotyping

All patients had continuous arterial pressure monitoring using a radial artery catheter. Based on the recorded arterial pressure waveforms we retrospectively computed haemodynamic variables using the Hypotension Prediction Index (HPI) software library (Edwards Lifesciences; Irvine, CA, USA).^8^^,^^14^ Specifically, we determined mean arterial pressure (MAP), stroke volume index (SVI), heart rate (HR), cardiac index (CI), systemic vascular resistance index (SVRI) (for which central venous pressure was assumed to be 5 mm Hg), stroke volume variation (SVV), and the maximum value of the first derivative of pressure with respect to time (dP/dt). For each haemodynamic variable, we extracted 20-s average data points. Poor quality arterial waveform signals were detected by the arterial beat detection algorithm of the HPI software library and excluded from analysis.^8^^,^^14^

We defined hypotension as a MAP <65 mm Hg for at least 1 min.^15^^,^^16^ To identify and validate endotypes of hypotension we considered input variables that are related to hypotension and clinically actionable. SVI, HR, CI, and SVRI were logical primary candidate variables. However, CI is redundant with SVI and HR. To account for the dependency of SVI on cardiac preload and myocardial contractility we considered including dP/dt (as a measure of left ventricular contractility)^17^ and SVV (as a dynamic cardiac preload variable predicting fluid responsiveness).^18^ During development of the unsupervised deep learning model, including dP/dt did not further improve the model for endotyping. Therefore, we finally considered SVI, HR, SVRI, and SVV as input variables for the unsupervised deep learning model to identify endotypes of hypotension.

### Identification of hypotension endotypes in the development dataset

**Fig 1:**
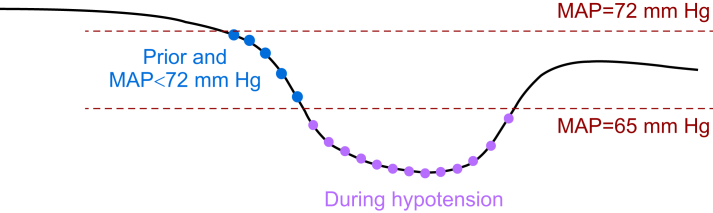
Illustration of data points during hypotension and immediately preceding hypotension. Hypotension was defined as a mean arterial pressure (MAP) <65 mm Hg for at least 1 minute. The figure illustrates data points observed during episodes of hypotension (purple) and data points observed during periods with a MAP <72 mm Hg immediately preceding episodes of hypotension (blue). Both purple and blue data points were used in the analyses.

We considered SVI, HR, SVRI, and SVV observed both during episodes of hypotension and during periods with a MAP <72 mm Hg immediately preceding episodes of hypotension (Fig. 1).^19^^,^^20^ As endotypes for actual hypotension (MAP <65 mm Hg) might differ from those during periods immediately preceding hypotension, we additionally performed endotyping considering only SVI, HR, SVRI, and SVV during episodes of hypotension (without considering periods with a MAP <72 mm Hg immediately preceding episodes of hypotension).

We used unsupervised deep learning to identify hypotension endotypes based on the input variables SVI, HR, SVRI, and SVV in the development dataset. Firstly, we normalised all SVI, HR, SVRI, and SVV values to a mean of 0 and a standard deviation of 1 to give each variable the same potential weight on the results. We then developed an autoencoder model, an artificial deep neural network, to encode data into a lower, two-dimensional latent space representation (Supplementary Fig. S1). The purpose of the autoencoder model is to learn an efficient representation of the data to reduce the dimensionality and noise of the data to make the identification of hypotension endotypes more robust and less subject to outliers. The learning curves of the autoencoder model are shown in Supplementary Figure S2.

A Gaussian mixture model (GMM), an unsupervised machine learning method, was then used to cluster data in the latent space into hypotension endotypes. The GMM is a probabilistic model that assumes data points come from a mixture of multiple Gaussian distributions, with each representing a cluster. It provides a probability score for each cluster and has the advantage of clustering data even when they are of different shapes and sizes.

To determine the optimal number of clusters (i.e. hypotension endotypes), two clustering evaluation metrics were used: the Calinski–Harabasz index^21^ evaluates the ratio of between-cluster variance and within-cluster variance and assesses the compactness and separability of clusters, and the Davies–Bouldin index^22^ measures the average similarity between each cluster and its most similar cluster and quantifies how well separated clusters are from each other. The Calinski–Harabasz index ranges from 0 to infinity, and a higher Calinski–Harabasz index indicates better clustering. The Davies–Bouldin index ranges from 0 to 1, and a lower Davies–Bouldin index indicates better clustering.

As a sensitivity analysis, we used k-means clustering, another unsupervised machine learning method, and compared the hypotension endotypes identified using the GMM method and the k-means method. The k-means method is based on slightly different assumptions, assuming clusters are spherical and of similar size.

### Validation of the hypotension endotypes in two independent validation datasets

The unsupervised deep learning model from the development dataset was validated in two independent datasets. Firstly, we aimed to validate whether unsupervised deep learning identifies the same number of endotypes in an independent dataset and whether the identified endotypes are similar to those identified in the development dataset. We thus repeated the identification of hypotension endotypes in the independent validation dataset including 1000 surgical patients and assessed the similarity between these endotypes and those identified in the development dataset. Secondly, we aimed to validate whether the unsupervised deep learning model trained from the development dataset including surgical patients generates similar hypotension endotypes in an independent dataset including critically ill patients. We thus applied the unsupervised deep learning model from the development dataset to the second independent validation dataset including 1000 critically ill patients, and assessed the similarity between the identified hypotension endotypes and those identified in the development dataset.

### Similarity of hypotension endotypes

To assess similarities between the hypotension endotypes identified using different methods and different data in the development dataset and the two independent validation datasets we used a measure called Kullback–Leibler divergence.^23^ The Kullback–Leibler divergence measures how one probability distribution (one hypotension endotype) diverges from a second probability distribution (another hypotension endotype). The Kullback–Leibler divergence ranges between 0 and infinity. The closer the Kullback–Leibler divergence is to 0, the more similar the two distributions are. If it is 0, the two distributions are identical.

### Statistical analysis

We used the Python programming language version 3.9.13 (Python Software Foundation, https://www.python.org/) and MATLAB version 2022b (The MathWorks, Natick, MA, USA) for statistical analyses.

## Results

We analysed >2 million data points from almost 70 000 hypotensive events in 2871 surgical or critically ill patients. We describe and quantify the amount of hypotension patients had and the haemodynamic variables used for endotyping in Supplementary Table S1.

### Hypotension endotypes in the development dataset

**Fig 2:**
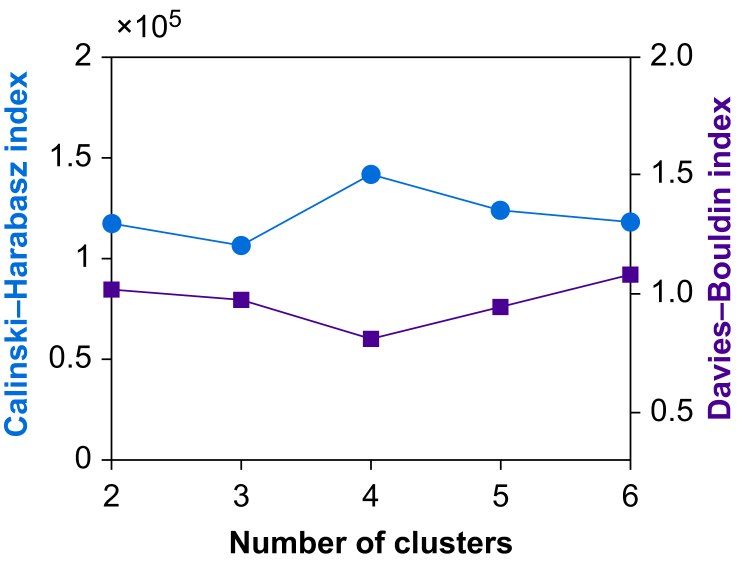
Optimal number of hypotension endotypes. Calinski–Harabasz index (blue circles) and Davies–Bouldin index (purple squares) for different numbers of clusters to determine the optimal number of clusters (i.e. hypotension endotypes) in the development dataset using a Gaussian mixture model in the autoencoder latent space. The plot shows that four is the optimal number of hypotension endotypes (highest Calinski–Harabasz index and lowest Davies–Bouldin index).

**Fig 3:**
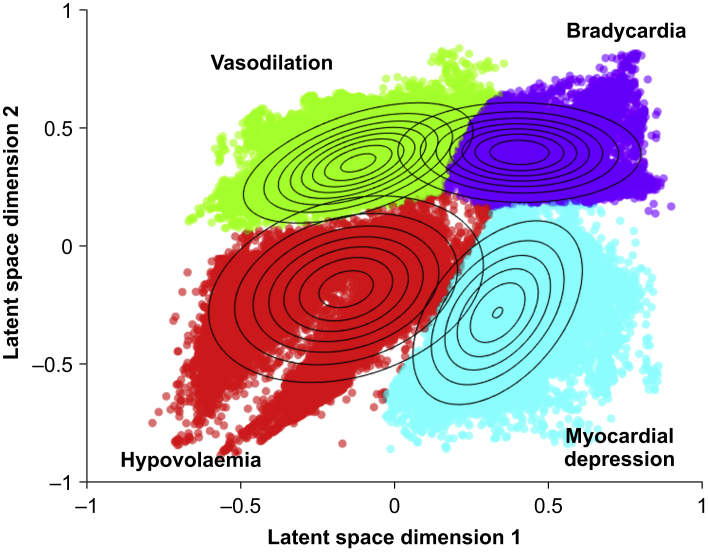
Endotypes of hypotension. This figure shows a two-dimensional autoencoder latent space representation of the data points clustered into the four endotypes (vasodilation, hypovolaemia, myocardial depression, and bradycardia) using a Gaussian mixture model in the development dataset (surgical patients). The circular rings overlaying the four endotypes represent the probability of the data points to belong to an endotype, with inner circles indicating a higher probability.

**Fig 4:**
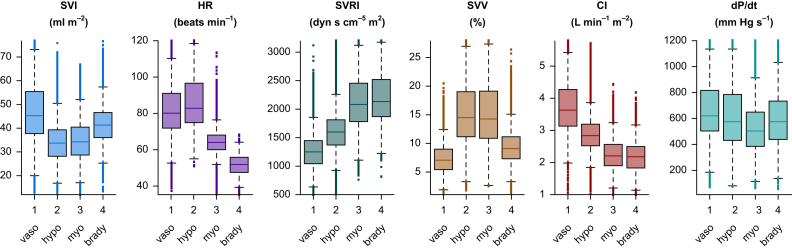
Non-normalised haemodynamic variables of the four hypotension endotypes. Boxplots showing non-normalised values of stroke volume index (SVI), heart rate (HR), systemic vascular resistance index (SVRI), stroke volume variation (SVV), cardiac index (CI), and the maximum value of the first derivative of pressure with respect to time (dP/dt) of data points in the development dataset for the four hypotension endotypes clustered with the Gaussian mixture model, namely vasodilation (vaso), hypovolaemia (hypo), myocardial depression (myo), and bradycardia (brady).

In the development dataset of 871 surgical patients, four was the optimal number of hypotension endotypes for GMM clustering when data points during episodes of hypotension and during periods with a MAP <72 mm Hg immediately preceding episodes of hypotension were modelled (Fig. 2). Based on their haemodynamic characteristics, we labelled the four endotypes as (1) vasodilation; (2) hypovolaemia; (3) myocardial depression; and (4) bradycardia (Table 1, Fig 3, Fig 4, and Supplementary Fig. S3). The most common endotype was vasodilation, contributing 54 774 of 156 857 (35%) hypotensive data points. The next most common endotype was hypovolaemia, contributing 50 295 of 156 857 (32%) hypotensive data points. Myocardial depression contributed 35 209 of 156 857 (22%), and bradycardia contributed 16 579 of 156 857 (11%) hypotensive data points (Supplementary Table S1).

**Table 1:** Endotypes of hypotension. Haemodynamic characteristics of the hypotension endotypes in the development dataset (surgical patients), the first validation dataset (surgical patients), and the second validation dataset (critically ill patients) identified using a Gaussian mixture model. Data are presented as absolute number (percentage) or mean (sd) and median (25th percentile–75th percentile). CI, cardiac index; dP/dt, maximum value of the first derivative of pressure with respect to time; HR, heart rate; SVI, stroke volume index; SVRI, systemic vascular resistance index; SVV, stroke volume variation.

Dataset	Endotype	*n* (%)	SVI (ml m^−2^)	HR (beats min^−1^)	SVRI (dyn s cm^−5^ m^2^)	SVV (%)	CI (L min^−1^ m^−2^)	dP/dt (mm Hg s^−1^)
Development dataset (surgical patients)	Vasodilation	54 774 (34.9)	47 (13), 45 (38–55)	82 (15), 80 (72–91)	1253 (296), 1253 (1044–1450)	7 (3), 7 (5–9)	3.8 (0.9), 3.6 (3.1–4.3)	682 (258), 620 (500–815)
	Hypovolaemia	50 295 (32.1)	34 (8), 34 (28–39)	87 (16), 83 (75–97)	1610 (342), 1601 (1375–1821)	16 (6), 14 (11–19)	2.9 (0.5), 2.8 (2.5–3.2)	629 (275), 573 (430–783)
	Myocardial depression	35 209 (22.4)	35 (9), 34 (29–40)	64 (7), 64 (60–68)	2268 (957), 2081 (1776–2448)	16 (6), 14 (11–19)	2.2 (0.6), 2.2 (1.9–2.6)	534 (227), 502 (382–648)
	Bradycardia	16 579 (10.6)	42 (9), 41 (36–47)	52 (5), 52 (47–56)	2255 (579), 2133 (1867–2517)	9 (3), 9 (7–11)	2.2 (0.5), 2.2 (1.8–2.5)	590 (203), 574 (435–733)
First validation dataset (surgical patients)	Vasodilation	46 513 (29.2)	47 (12), 46 (40–53)	92 (14), 89 (82–100)	1130 (351), 1111 (935–1300)	8 (3), 8 (6–10)	4.3 (1.2), 4.1 (3.5–4.8)	731 (263), 689 (565–850)
	Hypovolaemia	48 957 (30.7)	35 (10), 35 (29–41)	86 (15), 83 (75–93)	1591 (442), 1570 (1308–1848)	16 (7), 15 (12–19)	3.0 (0.8), 2.8 (2.5–3.4)	627 (270), 581 (449–751)
	Myocardial depression	33 740 (21.1)	34 (10), 34 (28–40)	65 (8), 64 (60–69)	2289 (908), 2116 (1772–2526)	16 (7), 14 (11–18)	2.2 (0.6), 2.2 (1.8–2.6)	591 (236), 563 (427–724)
	Bradycardia	30 327 (19.0)	53 (16), 51 (42–61)	57 (10), 55 (50–64)	1705 (628), 1589 (1259–2080)	8 (3), 8 (6–10)	3.1 (1.6), 2.9 (2.2–3.6)	666 (258), 628 (497–809)
Second validation dataset (critically ill patients)	Vasodilation	753 091 (39.6)	40 (14), 37 (30–47)	94 (18), 92 (83–103)	1312 (360), 1312 (1049–1547)	8 (3), 8 (6–10)	3.7 (1.1), 3.4 (2.9–4.2)	768 (347), 698 (522–954)
	Hypovolaemia	908 098 (47.8)	31 (8), 29 (25–35)	93 (14), 91 (83–100)	1648 (394), 1629 (1398–1866)	17 (6), 16 (12–20)	2.8 (0.7), 2.7 (2.4–3.1)	582 (277), 529 (384–735)
	Myocardial depression	199 611 (10.5)	32 (12), 33 (25–40)	68 (11), 66 (61–72)	2518 (1402), 2120 (1778–2633)	18 (8), 16 (12–21)	2.1 (0.7), 2.1 (1.7–2.5)	658 (339), 602 (423–857)
	Bradycardia	41 123 (2.1)	41 (9), 40 (34–46)	53 (6), 53 (50–56)	2223 (589), 2167 (1824–2550)	9 (4), 9 (7–11)	2.2 (0.5), 2.1 (1.8–2.5)	985 (546), 835 (604–1208)

For k-means clustering in the autoencoder latent space, four was the optimal number of hypotension endotypes (Supplementary Fig. S4). Based on their haemodynamic characteristics, the four endotypes could again be labelled as (1) vasodilation; (2) hypovolaemia; (3) myocardial depression; and (4) bradycardia. Kullback–Leibler divergence analysis showed that hypotension endotypes identified by GMM clustering and k-means clustering were very similar (Supplementary Table S2).

GMM clustering considering only data points during episodes of hypotension (without considering periods with a MAP <72 mm Hg immediately preceding episodes of hypotension), identified the same four hypotension endotypes (Supplementary Table S3). Kullback–Leibler divergence analysis showed that hypotension endotypes identified by GMM clustering were very similar when considering episodes of hypotension and periods with a MAP <72 mm Hg immediately preceding episodes of hypotension *vs* considering only episodes of hypotension (Supplementary Table S4).

### Hypotension endotypes in the validation datasets

In the first validation dataset of 1000 surgical patients, four was the optimal number of hypotension endotypes (Supplementary Fig. S5). Based on their haemodynamic characteristics, the four endotypes could again be labelled as (1) vasodilation; (2) hypovolaemia; (3) myocardial depression; and (4) bradycardia (Table 1, Supplementary Table S1, Supplementary Fig. S6). Kullback–Leibler divergence analysis showed that the hypotension endotypes identified in the first validation dataset and those identified in the development dataset were similar (Table 2).

**Table 2:** Similarity between hypotension endotypes in different datasets. Similarity between hypotension endotypes identified in the development dataset (surgical patients), the first validation dataset (surgical patients), and the second validation dataset (critically ill patients) using a Gaussian mixture model quantified by Kullback–Leibler divergence. The closer the Kullback–Leibler divergence is to 0, the more similar two hypotension endotypes are.

		Development dataset (surgical patients)			
		Vasodilation	Hypovolaemia	Myocardial depression	Bradycardia
First validation dataset (surgical patients)	Vasodilation	2.5	207.1	408.7	149.9
	Hypovolaemia	34.2	0.1	33.4	50.0
	Myocardial depression	294.6	185.0	0.07	30.3
	Bradycardia	21.9	249.8	250.6	0.3
Second validation dataset (critically ill patients)	Vasodilation	0.5	307.4	450.5	158.7
	Hypovolaemia	79.2	0.3	81.3	138.6
	Myocardial depression	346.6	202.8	0.1	44.9
	Bradycardia	95.6	334.2	289.1	0.2

When the unsupervised deep learning model from the development dataset was applied to the second independent validation dataset of 1000 critically ill patients, the same four hypotension endotypes were generated (Table 1, Supplementary Table S1). Kullback–Leibler divergence analysis showed that the hypotension endotypes identified in the second validation dataset and those identified in the development dataset were again similar (Table 2).

## Discussion

In this analysis of haemodynamic data from 2871 surgical or critically ill patients, unsupervised deep learning identified four endotypes of hypotension: vasodilation, hypovolaemia, myocardial depression, and bradycardia. We primarily used GMM clustering in the autoencoder latent space to identify hypotension endotypes because it is a robust method less susceptible to data noise and outliers. As a sensitivity analysis, we used a different clustering method (k-means) and identified the same four hypotension endotypes, which suggests that our results are robust. Additionally, unsupervised deep learning identified the same four hypotension endotypes in all three independent datasets including data of heterogenous surgical and critically ill patients from many hospitals and countries.

Hypotension is most effectively treated based on its underlying causes and mechanisms. However, in perioperative and intensive care medicine, specific causes of hypotension often remain unclear. Consequently, fluids and vasopressors remain the mainstay treatments.^24^ Considering hypotension endotypes might provide a more detailed understanding of the underlying haemodynamic alterations and allow causally treating hypotension with specific interventions. Clinicians could thus use hypotension endotypes as a guide to the intervention that is most likely to be effective to treat a given episode of hypotension in a given patient.

The endotypes we identified in the large datasets of surgical and critically ill patients using unsupervised deep learning are similar to those we previously found using traditional unsupervised machine learning in a single-centre study of 100 patients having major abdominal surgery.^7^ In that study, hierarchical clustering revealed six instead of four hypotension endotypes because it differentiated two subtypes of the vasodilation endotype and classified certain hypotensive episodes as mixed endotype.^7^ Including more patients and using more sophisticated and robust methods to cluster hypotensive episodes allowed classifying hypotensive episodes into one of four distinct hypotension endotypes.

The hypotension endotypes we identified using unsupervised deep learning are physiologically and clinically plausible. When advanced haemodynamic variables are available, clinicians could identify hypotension endotypes themselves using simple basic rules and predefined cut-off values of haemodynamic variables without using artificial intelligence. However, clustering haemodynamic variables into hypotension endotypes in real time at the bedside can be difficult, and hypotension endotypes can interact, overlap, and rapidly change. Unsupervised deep learning decision support thus could help automatically identify hypotension endotypes in real time. Additionally, there can be different underlying causes of hypotension at the same time. Our study forms the basis for artificial intelligence-assisted real-time haemodynamic guidance. Specifically, hypotension endotypes could be incorporated into monitoring systems informing haemodynamic management and providing clinical decision support.

The advantage of our algorithm is that it not only can identify the four hypotension endotypes, but it can also provide the probability of each endotype for each hypotensive data point because each Gaussian component in the GMM has its own probability density function characterised by a mean and a covariance matrix. These probability density functions define the likelihood of a data point belonging to each Gaussian component. Thus, GMM can provide the probability of each data point belonging to different endotypes because of its probabilistic framework. As there can be more than one underlying cause of hypotension, knowing the probability of each underlying cause might help clinicians select and prioritise therapeutic interventions (Supplementary Fig. S7).

There is no clear definition for hypotension in surgical^25^ or critically ill^26^ patients. Hypotension was thus pragmatically defined as a MAP <65 mm Hg in this analysis. From a pathophysiological perspective, hypotension means that arterial pressure is too low to ensure optimal organ perfusion. Most organs autoregulate their blood flow across varying perfusion pressures.^27^ For a single organ, hypotension occurs when arterial pressure is below the lower limit of autoregulation, which is usually unknown in clinical practice. We pragmatically defined hypotension as a MAP <65 mm Hg because numerous registry studies show that at a population level the MAP harm threshold for hypoperfusion-related organ injury, such as acute kidney injury or myocardial injury, is 60–70 mm Hg.^1^, ^2^, ^3^^,^^28^ We aimed to identify not only endotypes during hypotension, but also to identify endotypes when patients are developing hypotension. We thus considered data points before hypotension occurred (i.e. when MAP was <72 mm Hg). The MAP threshold of 72 mm Hg comes from the finding that when MAP is <72 mm Hg, there is a reasonably high chance that it decreases further to <65 mm Hg.^19^^,^^20^

Haemodynamic variables are closely interrelated, and hence hypotension endotypes can overlap and might not be categorically defined. From a methodological perspective, hypotensive data points can belong to multiple endotypes, therefore the assigned endotype represents the probability that hypotension is caused by one of the four underlying haemodynamic alterations.

Our analyses do not consider the temporal relationship among hypotensive episodes. We evaluated multiple data points within single episodes of hypotension and multiple episodes of hypotension within individual patients, treating data points as if they were independent, although they are not. A patient can stay within a certain hypotension endotype or can move from one hypotension endotype to another as a function of time. These aspects merit further analysis, especially for real-time identification of hypotension endotypes.

Our study has additional limitations. We only included patients who had continuous arterial pressure monitoring with an arterial catheter. Future research should evaluate whether considering haemodynamic data from sources other than arterial pressure waveforms, for example data from other sensors, physical examination, or laboratory tests, can help refine hypotension endotypes. We analysed >2 million hypotensive data points in almost 3000 surgical and critically ill patients. Hypotension endotypes reflect basic cardiovascular physiology, and it is thus likely that hypotension endotypes are highly consistent across different patient populations. However, while the endotypes are a robust finding, the relative frequency of the endotypes will naturally differ among different patient populations and settings. We used SVV as an input variable. SVV cannot reliably predict fluid responsiveness in patients with spontaneous breathing activity or cardiac arrythmias.^29^ However, we did not use SVV to predict fluid responsiveness but as a general input variable to identify endotypes. Cardiac arrhythmias that were too severe to be handled by the SVV algorithm,^30^ such as atrial fibrillation, were removed from the analyses. Therefore, further research is needed to determine if and how well the unsupervised deep learning algorithm can identify hypotension endotypes in patients with severe cardiac arrhythmias. For SVRI calculation, central venous pressure was assumed to be 5 mm Hg, which is a good approximation most of the time, but at times central venous pressure can deviate from 5 mm Hg. Finally, in this analysis we identified and validated four hypotension endotypes. Future studies are warranted to investigate whether causally treating hypotension considering the specific hypotension endotype improves patient-centred outcomes.

### Conclusions

Unsupervised deep learning identified four endotypes of hypotension in surgical and critically ill patients: vasodilation, hypovolaemia, myocardial depression, and bradycardia. The algorithm provides the probability of each endotype for each hypotensive data point. Identifying hypotension endotypes could guide clinicians to causal treatments for hypotension. Clinical trials are needed to investigate whether endotype-guided causal treatment of hypotension improves patient-centred outcomes.

## Authors’ contributions

Study conception and design: ZJ, FH, BS

Study measurements: ZJ, FH

Algorithm development: ZJ, XL

Data collection: ZJ, JS, SD, TWLS, NWF, DPV, APJV, MS, MC, DB, MRP, DIS, FH

Data interpretation: all authors

Statistical analysis: ZJ, XL

Drafting of the manuscript: ZJ, JS, FH, BS

Critical revision of the manuscript for important intellectual content and final approval of the version to be published: all authors

Agreement to be accountable for all aspects of the work thereby ensuring that questions related to the accuracy or integrity of any part of the work are appropriately investigated and resolved: all authors

Had full access to all data in the study and are responsible for the integrity of the data and the accuracy of the data analysis: ZJ, XL

## Funding

Supported solely by Edwards Lifesciences (Irvine, CA, USA) and the institutions and departments of the authors. No Edwards employees other than those included as authors were involved in directing the design of the study, the decision to publish, or approval of the manuscript.

## Declarations of interest

ZJ, XL, JS, TWLS, FH are employees of Edwards Lifesciences (Irvine, CA, USA). KK is a consultant for and has received honoraria for giving lectures from Edwards Lifesciences Inc. (Irvine, CA, USA). KK is a consultant for Vygon (Aachen, Germany). SD is a consultant for Edwards Lifesciences (Irvine, CA, USA) and has received research funding from the company. NWF has supported contracted research for Acacia Pharma, Tsumura Pharmaceuticals, Haisco Pharmaceuticals, Masimo, and Edwards Lifesciences and received honoraria from Edwards Lifesciences for invited presentations. DPV is a consultant for Edwards Lifesciences (Irvine, CA, USA) and has received research funding from the company and from Philips Medical BV. APJV received consulting, lecturing fees and grants by Edwards Lifesciences paid to the institution. MS is consultant for Edwards Lifesciences and has received institutional research funding for investigator-initiated trials and honoraria for giving lectures from Edwards Lifesciences, has received honoraria for giving lectures from AMOMED (Vienna, Austria), and has received honoraria for giving lectures from Orion Pharma (Hamburg, Germany). MS has received honoraria for giving lectures from Philips Medizin Systeme Böblingen (Böblingen, Germany). MC is a consultant for Edwards Lifesciences and Masimo Corp., and has funded research from Edwards Lifesciences and Masimo Corp. He is also the founder of Sironis and Perceptive Medical and owns patents and receives royalties for closed loop haemodynamic management technologies that have been licensed to Edwards Lifesciences. DB received consulting fees and grants by Edwards Lifesciences paid to the institution. MRP was a consultant and received honoraria for giving lectures from Edwards Lifesciences (Irvine, CA, USA), Masimo Medical (Irvine, CA, USA), and Baxter Medical (Deerfield, IL, USA). DIS is a consultant for Edwards Lifesciences (Irvine, CA, USA) and has received research funding from the company; he has an equity interest in Perceptive Medical (Irvine, CA, USA). BS is a consultant for and has received institutional restricted research grants and honoraria for giving lectures from Edwards Lifesciences (Irvine, CA, USA). BS is a consultant for Philips North America (Cambridge, MA, USA) and has received honoraria for giving lectures from Philips Medizin Systeme Böblingen (Böblingen, Germany). BS has received institutional restricted research grants and honoraria for giving lectures from Baxter (Deerfield, IL, USA). BS is a consultant for and has received institutional restricted research grants and honoraria for giving lectures from GE Healthcare (Chicago, IL, USA). BS has received institutional restricted research grants and honoraria for giving lectures from CNSystems Medizintechnik (Graz, Austria). BS is a consultant for Maquet Critical Care (Solna, Sweden). BS has received honoraria for giving lectures from Getinge (Gothenburg, Sweden). BS is a consultant for and has received institutional restricted research grants and honoraria for giving lectures from Pulsion Medical Systems (Feldkirchen, Germany). BS is a consultant for and has received institutional restricted research grants and honoraria for giving lectures from Vygon (Aachen, Germany). BS is a consultant for and has received institutional restricted research grants from Retia Medical (Valhalla, NY, USA). BS has received honoraria for giving lectures from Masimo (Neuchâtel, Switzerland). BS is a consultant for Dynocardia (Cambridge, MA, USA). BS has received institutional restricted research grants from Osypka Medical (Berlin, Germany). BS received honoraria for giving lectures from Ratiopharm (Ulm, Germany). BS was a consultant for and has received institutional restricted research grants from Tensys Medical (San Diego, CA, USA). BS is an editor of the *British Journal of Anaesthesia*.

## References

[1] Ahuja S., Mascha E.J., Yang D. (2020). Associations of intraoperative radial arterial systolic, diastolic, mean, and pulse pressures with myocardial and acute kidney injury after noncardiac surgery: a retrospective cohort analysis. Anesthesiology.

[2] Gregory A., Stapelfeldt W.H., Khanna A.K. (2021). Intraoperative hypotension is associated with adverse clinical outcomes after noncardiac surgery. Anesth Analg.

[3] Wesselink E.M., Kappen T.H., Torn H.M., Slooter A.J.C., van Klei W.A. (2018). Intraoperative hypotension and the risk of postoperative adverse outcomes: a systematic review. Br J Anaesth.

[4] Maheshwari K., Nathanson B.H., Munson S.H. (2018). The relationship between ICU hypotension and in-hospital mortality and morbidity in septic patients. Intensive Care Med.

[5] Vincent J.L., Nielsen N.D., Shapiro N.I. (2018). Mean arterial pressure and mortality in patients with distributive shock: a retrospective analysis of the MIMIC-III database. Ann Intensiv Care.

[6] Khanna A.K., Maheshwari K., Mao G. (2019). Association between mean arterial pressure and acute kidney injury and a composite of myocardial injury and mortality in postoperative critically ill patients: a retrospective cohort analysis. Crit Care Med.

[7] Kouz K., Brockmann L., Timmermann L.M. (2023). Endotypes of intraoperative hypotension during major abdominal surgery: a retrospective machine learning analysis of an observational cohort study. Br J Anaesth.

[8] Hatib F., Jian Z., Buddi S. (2018). Machine-learning algorithm to predict hypotension based on high-fidelity arterial pressure waveform analysis. Anesthesiology.

[9] Davies S.J., Vistisen S.T., Jian Z., Hatib F., Scheeren T.W.L. (2020). Ability of an arterial waveform analysis-derived hypotension prediction index to predict future hypotensive events in surgical patients. Anesth Analg.

[10] Shin B., Maler S.A., Reddy K., Fleming N.W. (2021). Use of the hypotension prediction index during cardiac surgery. J Cardiothorac Vasc Anesth.

[11] Maheshwari K., Shimada T., Yang D. (2020). Hypotension prediction index for prevention of hypotension during moderate- to high-risk noncardiac surgery. Anesthesiology.

[12] Schneck E., Schulte D., Habig L. (2020). Hypotension prediction index based protocolized haemodynamic management reduces the incidence and duration of intraoperative hypotension in primary total hip arthroplasty: a single centre feasibility randomised blinded prospective interventional trial. J Clin Monit Comput.

[13] Wijnberge M., Geerts B.F., Hol L. (2020). Effect of a machine learning-derived early warning system for intraoperative hypotension vs standard care on depth and duration of intraoperative hypotension during elective noncardiac surgery: the HYPE randomized clinical trial. JAMA.

[14] Pratt B., Roteliuk L., Hatib F., Frazier J., Wallen R.D. (2007). Calculating arterial pressure-based cardiac output using a novel measurement and analysis method. Biomed Instrum Technol.

[15] Sessler D.I., Bloomstone J.A., Aronson S. (2019). Perioperative Quality Initiative consensus statement on intraoperative blood pressure, risk and outcomes for elective surgery. Br J Anaesth.

[16] Evans L., Rhodes A., Alhazzani W. (2021). Surviving sepsis campaign: international guidelines for management of sepsis and septic shock 2021. Intensive Care Med.

[17] Monge Garcia M.I., Jian Z., Settels J.J. (2018). Performance comparison of ventricular and arterial dP/dt(max) for assessing left ventricular systolic function during different experimental loading and contractile conditions. Crit Care.

[18] Zhang Z., Lu B., Sheng X., Jin N. (2011). Accuracy of stroke volume variation in predicting fluid responsiveness: a systematic review and meta-analysis. J Anesth.

[19] Mulder M.P., Harmannij-Markusse M., Fresiello L., Donker D.W., Potters J.W. (2024). Hypotension Prediction Index is equally effective in predicting intraoperative hypotension during non-cardiac surgery compared to a mean arterial pressure threshold: a prospective observational study. Anesthesiology.

[20] Davies S.J., Sessler D.I., Jian Z. (2024). Comparison of differences in cohort (forward) and case control (backward) methodological approaches for validation of the Hypotension Prediction Index. Anesthesiology.

[21] Caliński T., Harabasz J. (1974). A dendrite method for cluster analysis. Commun Stat.

[22] Davies D.L., Bouldin D.W. (1979). A cluster separation measure. IEEE Trans Pattern Anal Mach Intell.

[23] Kullback S., Leibler R.A. (1951). On information and sufficiency. Ann Math Stat.

[24] Chiu C., Fong N., Lazzareschi D. (2022). Fluids, vasopressors, and acute kidney injury after major abdominal surgery between 2015 and 2019: a multicentre retrospective analysis. Br J Anaesth.

[25] Weinberg L., Li S.Y., Louis M. (2022). Reported definitions of intraoperative hypotension in adults undergoing non-cardiac surgery under general anaesthesia: a review. BMC Anesthesiol.

[26] Cecconi M., De Backer D., Antonelli M. (2014). Consensus on circulatory shock and hemodynamic monitoring. Task force of the European Society of Intensive Care Medicine. Intensive Care Med.

[27] Meng L., Wang Y., Zhang L., McDonagh D.L. (2019). Heterogeneity and variability in pressure autoregulation of organ blood flow: lessons learned over 100+ years. Crit Care Med.

[28] Salmasi V., Maheshwari K., Yang D. (2017). Relationship between intraoperative hypotension, defined by either reduction from baseline or absolute thresholds, and acute kidney and myocardial injury after noncardiac surgery: a retrospective cohort analysis. Anesthesiology.

[29] Michard F. (2005). Changes in arterial pressure during mechanical ventilation. Anesthesiology.

[30] Cannesson M., Tran N.P., Cho M., Hatib F., Michard F. (2012). Predicting fluid responsiveness with stroke volume variation despite multiple extrasystoles. Crit Care Med.

