# Supplementary material for: Deep learning model to identify and validate hypotension endotypes in surgical and critically ill patients
Source: Br J Anaesth. 2025 Jan 8;134(2):308–16. doi: 10.1016/j.bja.2024.10.048 (PMC11775843; doi:10.1016/j.bja.2024.10.048)
Supplement: Multimedia component 1 [file mmc1.pdf]

Supplementary materials

Jian Z, Liu X, Kouz K, et al, Deep learning to identify and validate hypotension endotypes in surgical and critically ill patients

Supplementary Figure S1: Architecture of the autoencoder model

This figure illustrates the architecture of the autoencoder model. Input variables (stroke volume index, heart rate, systemic vascular resistance index, and stroke volume variation) are normalized, and encoded by the encoder to lower their dimensionality into two dimensions in the latent space. Encoded data are then clustered into endotypes using unsupervised learning, and the decoder layer decodes the clustered data in the latent space back to the original data space.

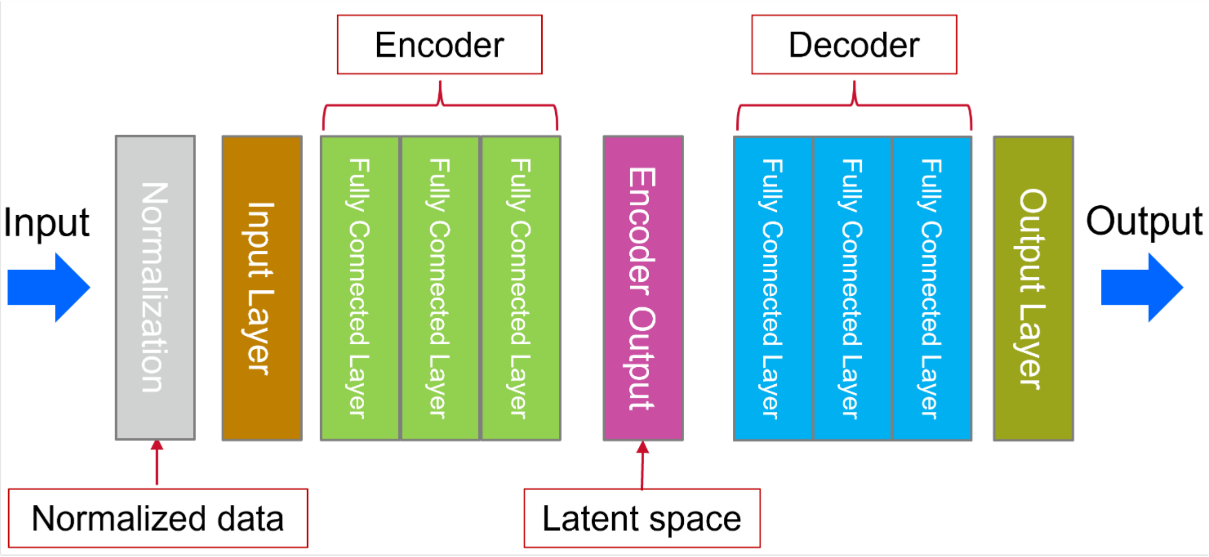

Supplementary Figure S2: Learning curves of the autoencoder model

This figure illustrates the learning curves of the autoencoder model for the training (red) and cross-validation (blue) sets.

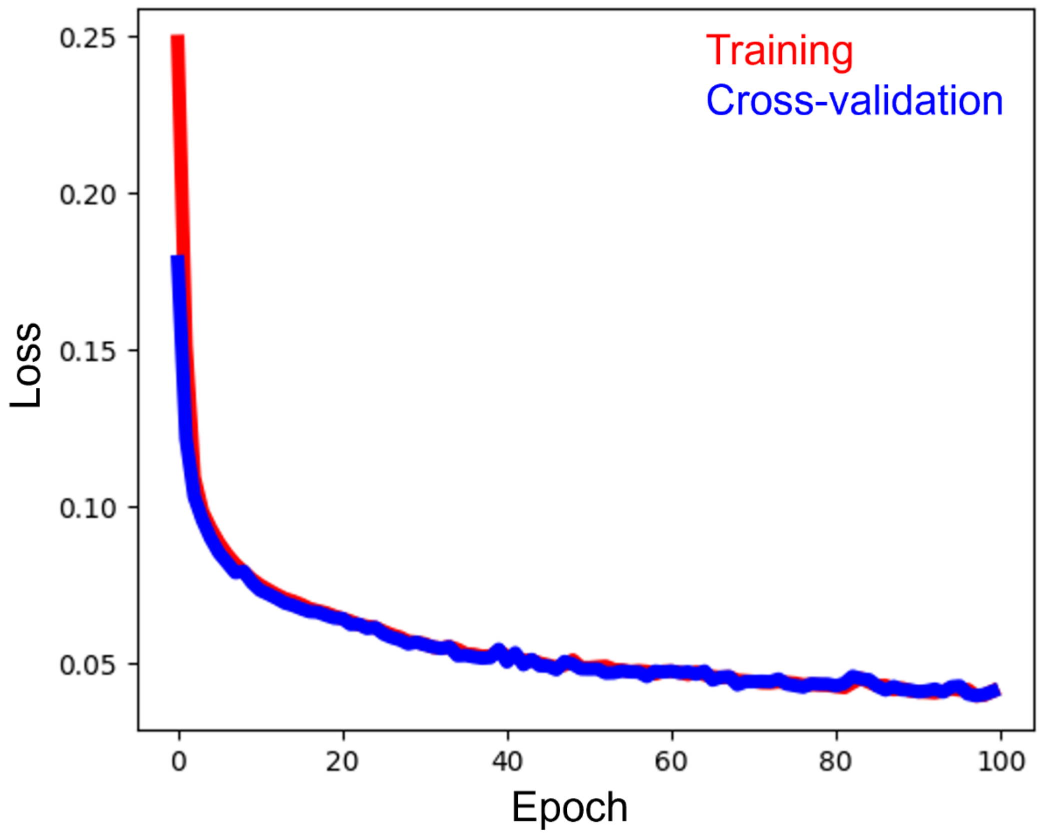

Supplementary Figure S3: Normalized hemodynamic variables of the four hypotension endotypes

Boxplots showing normalized values of stroke volume index (SVI), heart rate (HR), systemic vascular resistance index (SVRI), stroke volume variation (SVV), cardiac index (CI), and the maximum value of the first derivative of pressure with respect to time (dP/dt) for the four hypotension endotypes (Gaussian Mixture Model in the development dataset). Data are normalized to a mean of zero (dashed horizontal line) and a standard deviation of 1.

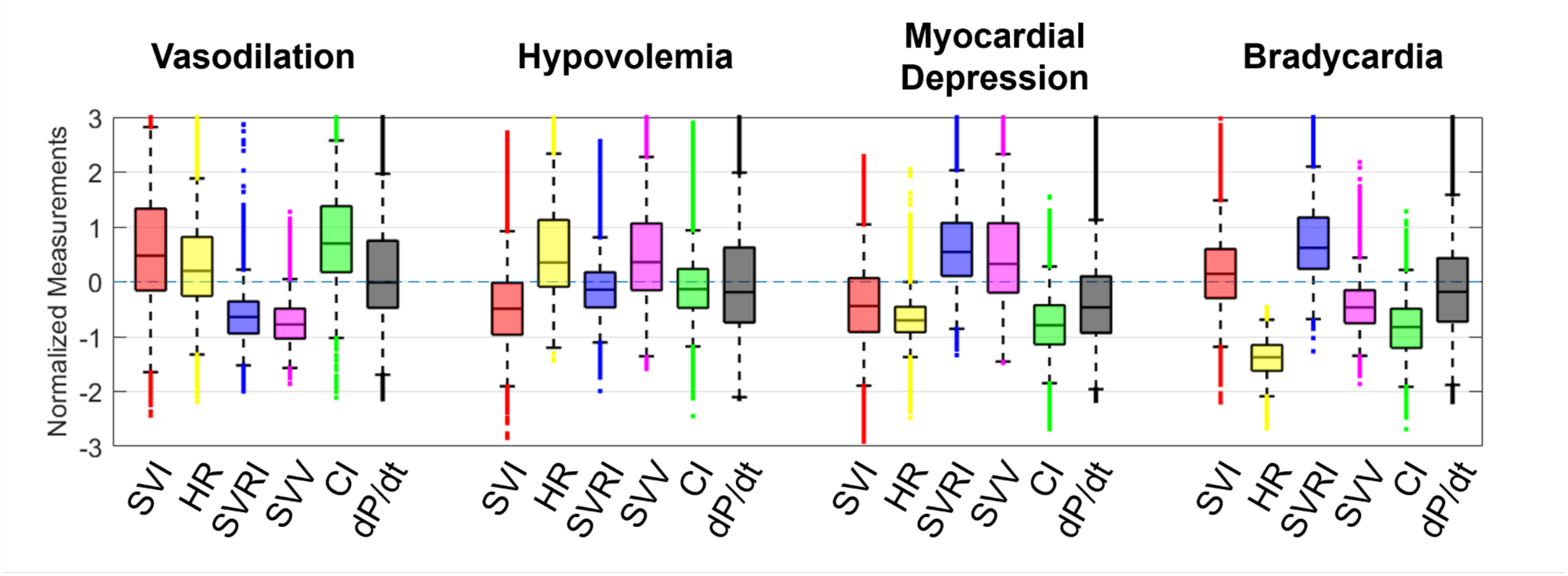

Supplementary Figure S4: Optimal number of hypotension endotypes (development dataset) using k-means clustering

Calinski-Harabasz index (blue) and Davies-Bouldin index (red) to determine the optimal number of clusters (i.e., hypotension endotypes) in the development dataset (surgical patients) using k-means clustering in the autoencoder latent space. The plot shows that four is the optimal number of hypotension endotypes (highest Calinski-Harabasz index and lowest Davies-Bouldin index).

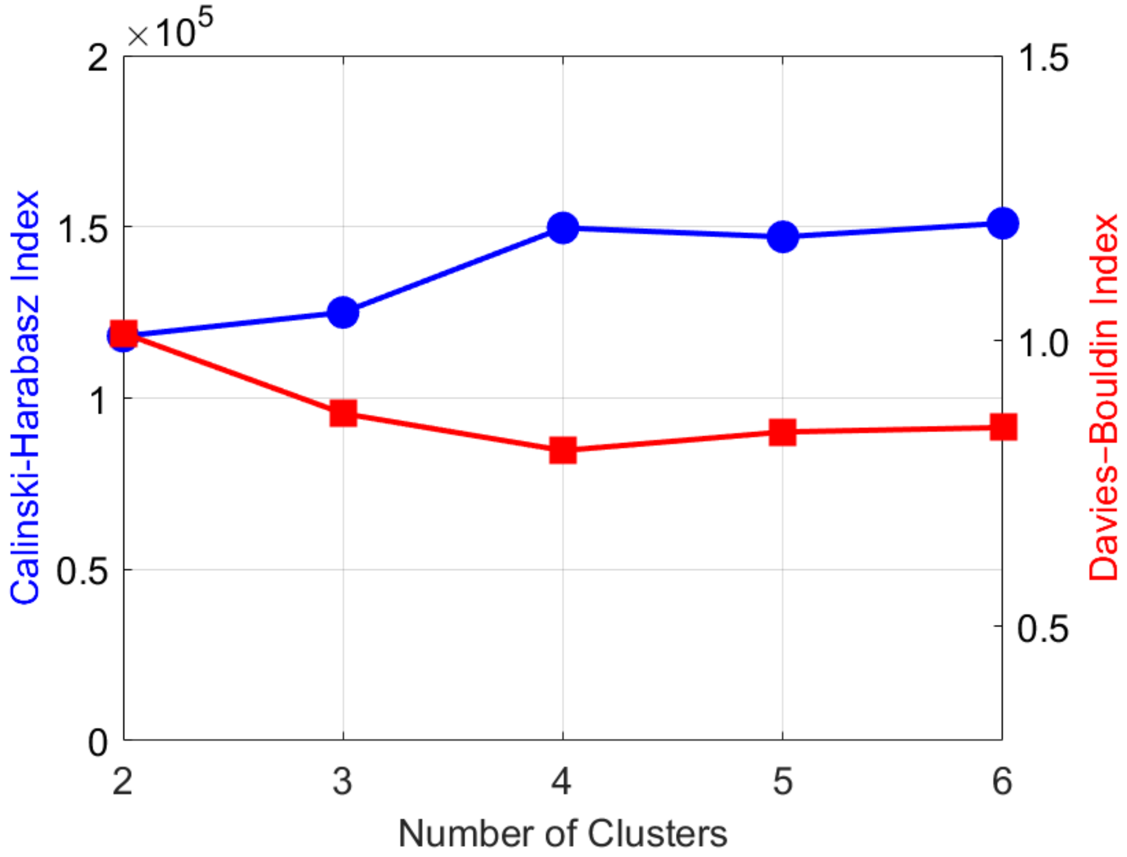

Supplementary Figure S5: Optimal number of hypotension endotypes (first validation dataset)

Calinski-Harabasz index (blue) and Davies-Bouldin index (red) to determine the optimal number of clusters (i.e., hypotension endotypes) in the first validation dataset (surgical patients) using a Gaussian Mixture Model in the autoencoder latent space. The plot shows that four is the optimal number of hypotension endotypes (highest Calinski-Harabasz index and lowest Davies-Bouldin index).

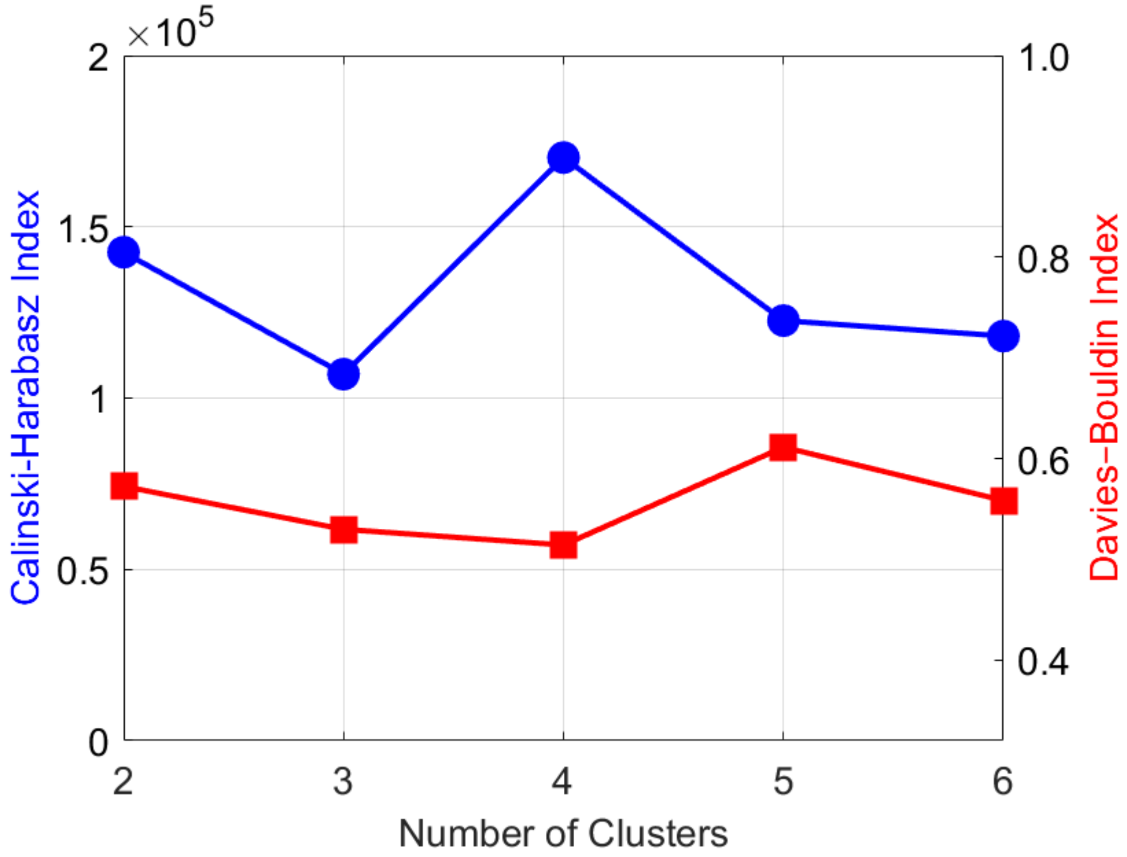

Supplementary Figure S6: Endotypes of hypotension

A two-dimensional autoencoder latent space representation of the data points clustered into the four endotypes (vasodilation, hypovolemia, myocardial depression, bradycardia) using a Gaussian Mixture Model in the first validation dataset (surgical patients). The circular rings overlaying the four endotypes represent the probability of the data points to belong to an endotype. The more inner the circle, the higher the probability for a data point to belong to the endotype.

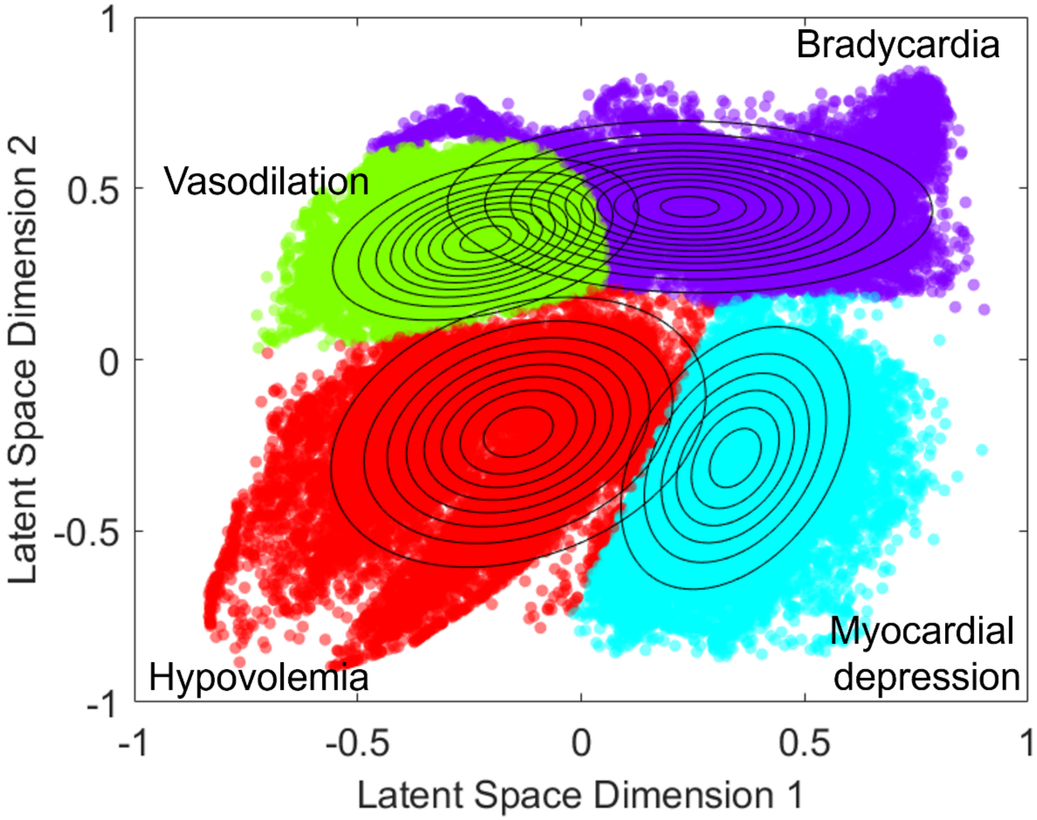

Supplementary Figure S7: Probability of hypotension endotypes for one patient

The figure exemplarily shows that the normalised probability of each hypotension endotype changes over time: First, bradycardia is the primary endotype, later the primary endotype is myocardial depression.

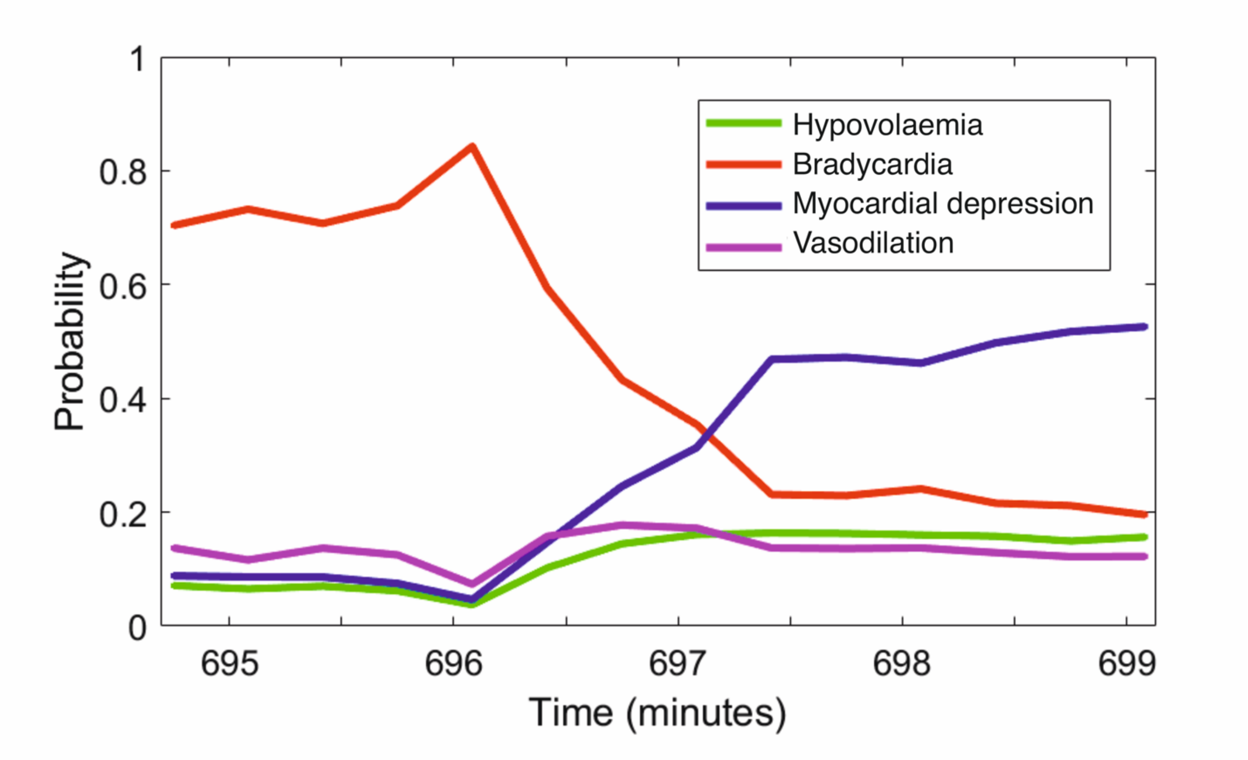

Supplementary Table S1: Patient characteristics, amount of hypotension, and hemodynamic monitoring data of the endotyping data points.

This was a retrospective analysis of three aggregated datasets including prospectively collected anonymized data of a total of 2,871 surgical or critically ill patients.

The development dataset, used for the development of the unsupervised deep learning model to identify hypotension endotypes, included 871 surgical patients from six previously reported studies [S1-S6]. All studies were approved by the respective ethics committees and – when the need for informed consent was not waived – patients provided written informed consent. Detailed inclusion and exclusion criteria are presented in the individual studies, and a summary is provided in this table.

The first independent validation dataset included 1,000 surgical patients who had surgery at the University of California Irvine Medical Center (Irvine, CA, USA) between 2015 and 2023. This observational data collection was approved by the ethics committee. The need for informed consent was waived. More than 1,000 patients were collected, and 1,000 patients were randomly selected for analyses.

The second independent validation dataset included 1,000 critically ill patients: 400 collected at the University of Pittsburgh Medical Center (Pittsburgh, PA, USA); 400 collected at the University Hospital of Bern (Bern, Switzerland); and 200 treated at the Beth Israel Deaconess Medical Center (Boston, MA, USA) who were included in the Medical Information Mart for Intensive Care (MIMIC) III database [S7]. The studies in Pittsburgh and Bern were approved by the ethics committee and individual informed consent was waived (Pittsburgh) or obtained (Bern). The MIMIC III database is publicly available and individual informed consent was waived. In each of the sites, more than 400 patients were collected, and 1,000 patients were randomly selected for analyses.

	Development dataset	First validation dataset of surgical patients	Second validation dataset of critically ill patients
Number of patients	871	1000	1000
Age (years)	63 (13),<br>65 (56, 72)	56 (18),<br>58 (45, 68)	64 (15),<br>66 (55, 75)
Height (cm)	170 (10),<br>170 (163, 178)	169 (11),<br>168 (160, 177)	172 (11),<br>173 (165,178)
Weight (kg)	81 (21),<br>80 (65, 91)	78 (22),<br>75 (63, 90)	86 (24),<br>83 (70, 98)
Sex (%)	Male 515 (59),<br>Female 356 (41)	Male 530 (53),<br>Female 470 (47)	Male 671 (67),<br>Female 329 (33)
American Society of Anesthesiologists physical status class (%)			
I	5 (1)	6 (1)	N/A
II	83 (10)	107 (11)	N/A
III	150 (17)	560 (56)	N/A
IV	22 (2)	242 (24)	N/A
V	0 (0)	29 (3)	N/A
VI	0 (0)	1 (0)	N/A
unknown	611 (70)	55 (5)	N/A
Patient population	- 52 patients having high risk non-cardiac surgeries [S1]<br>- 204 patients having major non-cardiac and cardiac surgery [S1]<br>- 255 patients having major non-cardiac and cardiac surgery [S2]<br>- 37 patients having elective cardiac surgery [S3]<br>- 213 patients having moderate-to-high-risk non-cardiac surgery [S4]<br>- 50 patients with total hip arthroplasty surgery [S5]<br>- 60 patients having elective non-cardiac surgery [S6]	- 118 patients having cardiac surgery<br>- 882 patients having non-cardiac surgery	- 400 critically ill patients (University of Pittsburgh Medical Center)<br>- 400 critically ill patients (University Hospital of Bern)<br>- 200 critically ill patients (Beth Israel Deaconess Medical Center)
**Amount of hypotension**			
Monitoring duration (min)	334 (386),<br>244 (171, 371)	299 (182),<br>261 (171, 402)	2,070 (2,652),<br>1,335 (852, 2106)
Number of patients with hypotension (%)	750 (86)	840 (84)	850 (85)

Total number of hypotensive events	6,962	7,904	5,3821
Average number of hypotensive events per patient	8 (13),<br>5 (1, 9)	8 (9),<br>5 (1, 12)	54 (85),<br>30 (5, 65)
Average duration of each hypotensive event (minutes)	5 (12),<br>2 (1, 5)	5 (10),<br>3 (1, 5)	9 (28),<br>2 (1, 6)
Cumulative duration of hypotensive events per patient (minutes)	40 (116),<br>12 (3, 37)	36 (53),<br>17 (3, 46)	487 (723),<br>271 (22, 728)
Cumulative duration of hypotensive events per patient (% of monitoring time)	11 (14),<br>5 (1, 14)	14 (24),<br>7 (2, 17)	31 (33),<br>18 (2, 55)
Area under a MAP of 65 mmHg per patient (mmHg*min)	263 (784),<br>72 (16, 219)	263 (754),<br>88 (17, 261)	3438 (6,087), 1<br>491 (111, 4650)
Time weighted average MAP <65mmHg per patient (mmHg)	0.71 (1.22),<br>0.30 (0.06, 0.83)	1.22 (3.7),<br>0.32 (0.07, 0.94)	2.36 (3.54),<br>0.84 (0.11, 3.44)
**Hemodynamic monitoring data used for endotyping**			
Number of endotyping data points (during hypotension and during periods with MAP <72 mmHg immediately preceding episodes of hypotension)	156,857	159,537	1,901,923
Number of endotyping data points (during hypotension)	90,203	89,851	1,298,707
Number of endotyping data points (during periods with MAP <72 mmHg immediately preceding episodes of hypotension)	66,654	69,686	603,216
Average duration of during periods with MAP <72 mmHg immediately	3 (4),<br>2 (1, 4)	3 (5),<br>2 (1, 4)	4 (9),<br>2 (1, 4)

preceding episodes of hypotension (min)			
SVI (ml m^{-2})	40 (12),<br>38 (32, 46)	42 (14),<br>40 (33, 49)	35 (12),<br>33 (27, 41)
HR (beats min^{-1})	76 (18),<br>75 (64, 87)	78 (19),<br>76 (64, 90)	90 (18),<br>90 (78, 100)
SVRI (dyn s cm^{-5} m^2)	1701 (697),<br>1590 (1283, 1972)	1626 (715),<br>1504 (1164, 1948)	1619 (685),<br>1541 (1259, 1836)
SVV (%)	12 (7),<br>11 (8, 15)	12 (7),<br>11 (8, 15)	13 (7),<br>12 (8, 17)
CI (L min^{-1} m^{-2})	3.0 (1.0),<br>2.9 (2.4, 3.5)	3.2 (1.3),<br>3.0 (2.4, 3.9)	3.1 (1.0),<br>2.9 (2.4, 3.5)
dP/dt (mmHg s^{-1})	622 (258),<br>573 (443, 758)	657 (264),<br>619 (484, 793)	672 (335),<br>606 (435, 842)

Data are presented as absolute number (percentage), mean (standard deviation), or median (25th percentile, 75th percentile).

N/A, not available.

Supplementary Table S2: Similarity between hypotension endotypes in the development dataset identified using Gaussian Mixture Model *versus* k-means clustering

Similarity between hypotension endotypes identified in the development dataset (surgical patients) using Gaussian Mixture Model *versus* k-means clustering quantified by Kullback-Leibler divergence. The closer the Kullback-Leibler divergence is to 0, the more similar two hypotension endotypes are.

		**Development dataset (Gaussian Mixture Model)**			
		**Vasodilation**	**Hypovolemia**	**Myocardial depression**	**Bradycardia**
**Development dataset (k-means clustering)**	**Vasodilation**	0.42	108.25	281.80	88.84
	**Hypovolemia**	60.35	0.39	61.27	142.80
	**Myocardial depression**	106.84	67.84	0.19	50.83
	**Bradycardia**	45.52	209.88	175.35	0.12

Supplementary Table S3: Incidence of hypotension endotypes using different models in different datasets.

Model	Dataset	Hypotension events, n	Data points, n	Vasodilation, n	Hypovolemia, n	Myocardial depression, n	Bradycardia, n
GMM from the development dataset (including episodes of hypotension and preceding hypotension)	Development (surgical patients)	6,962	156,857	54,774 (34.9)	50,295 (32.1)	35,209 (22.4)	16,579 (10.6)
k-means model from the development dataset (including episodes of hypotension and preceding hypotension)	Development (surgical patients)	6,962	156,857	51,648 (32.9)	42,798 (27.3)	40,783 (26.0)	21,628 (13.8)
GMM form the development dataset (including **only** episodes of hypotension)	Development (surgical patients)	6,962	90,203	27,957 (31.0)	24051 (26.7)	24,956 (27.6)	13,239 (14.7)
GMM from the first validation dataset (including episodes of hypotension and preceding hypotension)	First validation dataset (surgical patients)	7,904	159,537	46,513 (29.2)	48,957 (30.7)	33,740 (21.1)	30,327 (19.0)
GMM from the development dataset (including episodes of hypotension and preceding hypotension)	Second validation dataset (critically ill patients)	53,821	1,901,923	753,091 (39.6)	908,098 (47.8)	199,611 (10.5)	41,123 (2.1)

Data are presented as absolute number (percentage).

Supplementary Table S4: Similarity between hypotension endotypes in the development dataset

Similarity between hypotension endotypes identified in the development dataset (surgical patients) using a Gaussian Mixture Model when considering episodes of hypotension and periods with mean arterial pressure (MAP) <72 mmHg immediately preceding episodes *versus* considering only episodes of hypotension (without considering periods with MAP <72 mmHg immediately preceding episodes of hypotension) quantified by Kullback-Leibler divergence. The closer the Kullback-Leibler divergence is to 0, the more similar two hypotension endotypes are.

		**Development dataset (hypotension AND preceding hypotension data points)**			
		**Vasodilation**	**Hypovolemia**	**Myocardial depression**	**Bradycardia**
**Development dataset (only hypotension data points)**	**Vasodilation**	0.88	211.64	391.34	68.09
	**Hypovolemia**	29.79	0.47	65.83	82.32
	**Myocardial depression**	60.45	43.10	0.29	22.02
	**Bradycardia**	23.48	330.73	291.18	0.16

References

S1. Hatib F, Jian Z, Buddi S, et al. Machine-learning Algorithm to Predict Hypotension Based on High-fidelity Arterial Pressure Waveform Analysis. *Anesthesiology*. 2018;129(4):663-674.

S2. Davies SJ, Vistisen ST, Jian Z, Hatib F, Scheeren TWL. Ability of an Arterial Waveform Analysis-Derived Hypotension Prediction Index to Predict Future Hypotensive Events in Surgical Patients. *Anesth Analg*. 2020;130(2):352-359.

S3. Shin B, Maler SA, Reddy K, Fleming NW. Use of the Hypotension Prediction Index During Cardiac Surgery. *J Cardiothorac Vasc Anesth*. 2021;35(6):1769-1775.

S4. Maheshwari K, Shimada T, Yang D, et al. Hypotension Prediction Index for Prevention of Hypotension during Moderate- to High-risk Noncardiac Surgery. *Anesthesiology*. 2020;133(6):1214-1222.

S5. Schneck E, Schulte D, Habig L, et al. Hypotension Prediction Index based protocolized haemodynamic management reduces the incidence and duration of intraoperative hypotension in primary total hip arthroplasty: a single centre feasibility randomised blinded prospective interventional trial. *J Clin Monit Comput*. 2020;34(6):1149-1158.

S6. Wijnberge M, Geerts BF, Hol L, et al. Effect of a Machine Learning-Derived Early Warning System for Intraoperative Hypotension vs Standard Care on Depth and Duration of Intraoperative Hypotension During Elective Noncardiac Surgery: The HYPE Randomized Clinical Trial. *JAMA*. 2020;323(11):1052-1060.

S7. Johnson AEW, Pollard TJ, Shen L, et al. MIMIC-III, a freely accessible critical care database. *Sci Data*. 2016;3:160035.
