# Supplementary material for: Deep learning model to identify and validate hypotension endotypes in surgical and critically ill patients
Source: Br J Anaesth. 2025 Jan 8;134(2):308–16. doi: 10.1016/j.bja.2024.10.048 (PMC11775843; doi:10.1016/j.bja.2024.10.048)
Supplement: Multimedia component 2 [file mmc2.docx]

**Appendices:**

- **Supplementary Material Figure S1:** Architecture of the autoencoder model
- **Supplementary Material Figure S2:** Learning curves of the autoencoder model
- **Supplementary Material Figure S3:** Normalised haemodynamic variables of the four hypotension endotypes
- **Supplementary Material Figure S4:** Optimal number of hypotension endotypes (development dataset) using k-means clustering
- **Supplementary Material Figure S5:** Optimal number of hypotension endotypes (first validation dataset)
- **Supplementary Material Figure S6:** Endotypes of hypotension
- **Supplementary Material Figure S7:** Probability of hypotension endotypes for one patient
- **Supplementary Material Table S1:** Patient characteristics, amount of hypotension, and haemodynamic monitoring data of the endotyping data points.
- **Supplementary Material Table S2:** Similarity between hypotension endotypes in the development dataset identified using Gaussian Mixture Model *versus* k-means clustering
- **Supplementary Material Table S3:** Incidence of hypotension endotypes using different models in different datasets.
- **Supplementary Material Table S4:** Similarity between hypotension endotypes in the development dataset
